# Research Progress and Prospect of Nanoplatforms for Treatment of Oral Cancer

**DOI:** 10.3389/fphar.2020.616101

**Published:** 2020-12-17

**Authors:** Zhilong Zhao, Dan Li, Ziqi Wu, Qihui Wang, Zhangyu Ma, Congxiao Zhang

**Affiliations:** ^1^ Department of Stomatology, The First Hospital of Jilin University, Changchun, China; ^2^ Department of Cancer Center, The First Hospital of Jilin University, Changchun, China; ^3^ Nanyang Medical College, Nanyang, China

**Keywords:** nanoplatforms, drug delivery system, oral squamous cell carcinoma, treatment of oral cancer, tumor targeted therapy

## Abstract

Oral cancers refer to malignant tumors associated with high morbidity and mortality, and oral squamous cell carcinoma accounts for the majority of cases. It is an important part of head and neck, and oral cancer is one of the six most common cancers in the world. At present, the traditional treatment methods for oral cancer include surgery, radiation therapy, and chemotherapy. However, these methods have many disadvantages. In recent years, nanomedicine, the delivery of drugs through nanoplatforms for the treatment of cancer, has become a promising substitutive therapy. The use of nanoplatforms can reduce the degradation of the drug in the body and accurately deliver it to the tumor site. This minimizes the distribution of the drug to other organs, thereby reducing its toxicity and allowing higher drug concentration at the tumor site. This review introduces polymer nanoparticles, lipid-based nanoparticles, metal nanoparticles, hydrogels, exosomes, and dendrimers for the treatment of oral cancer, and discusses how these nanoplatforms play an anti-cancer effect. Finally, the review gives a slight outlook on the future prospects of nanoplatforms for oral cancer treatment.

## Introduction

**FIGURE 1:**
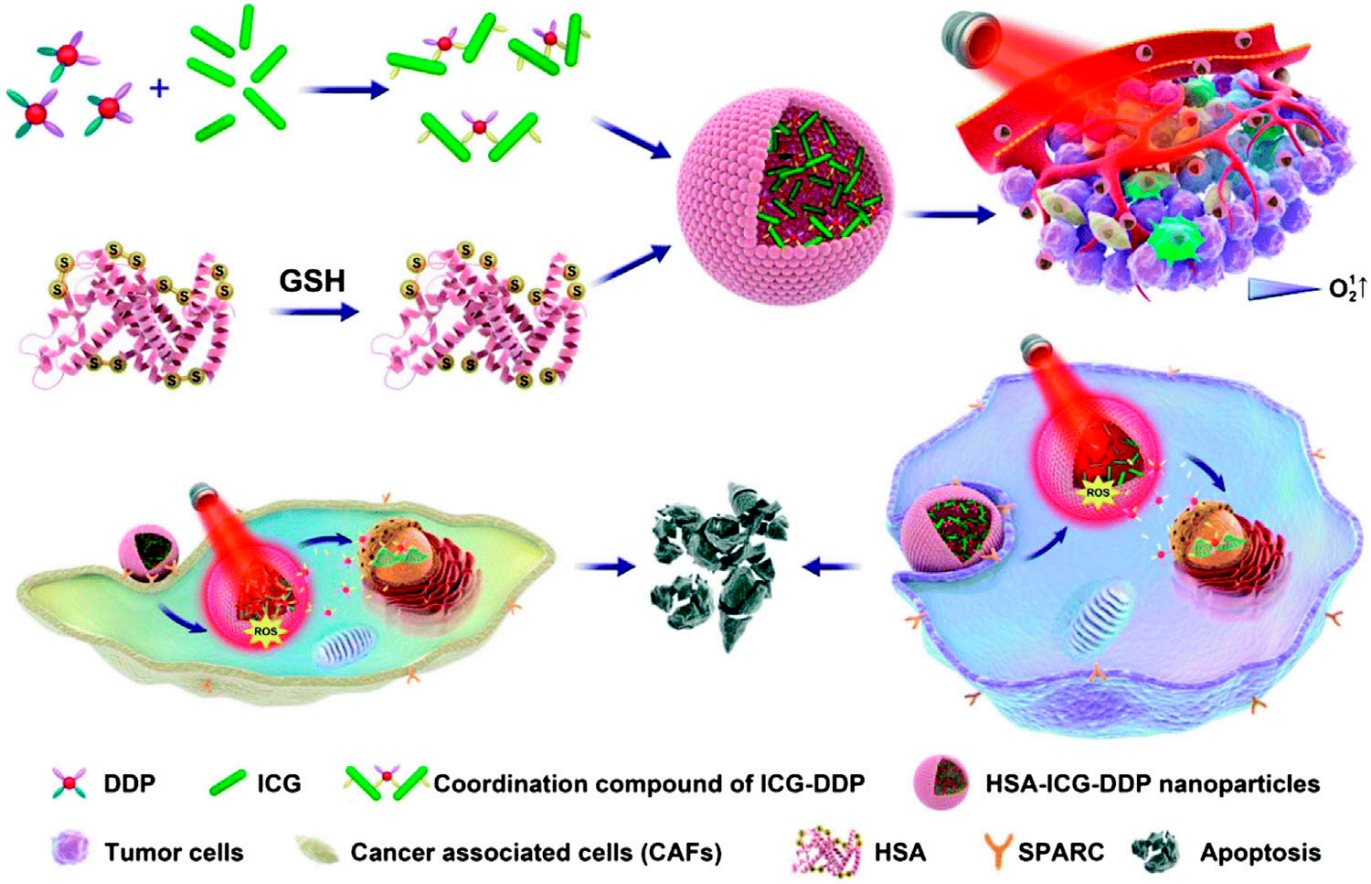
Schematic diagram of PDT/PTT/chemotherapy combination therapy triggered by NIR light. Reproduced from (Wang et al., 2019b) with permission from Biomaterials Science.

Oral squamous cell carcinoma (OSCC) is the main type of oral cavity tumor; it is associated with a poor prognosis, and the distant survival rate is <40% (Siegel et al., 2020). The oral cavity is an important site for head and neck tumors, and oral cancer is one of the six most common types of cancer worldwide (Siegel et al., 2013). Approximately two-thirds of patients with head and neck tumors have advanced disease (stages III and IV), and the high metastasis rate is closely related to the low 5-years survival rate (Woolgar et al., 1995; Noguti et al., 2012). Despite important advancements in various treatment modalities, such as surgery, chemotherapy, and radiotherapy, the long-term survival rate of patients with advanced head and neck tumors has not increased significantly over recent decades (Price and Cohen, 2012). In 2018, 354,864 head and neck tumors were diagnosed, and 177,384 individuals worldwide died due to these tumors (Jemal et al., 2011; Bray et al., 2018). The main risk factors for OSCC are shown in Figure 1. Smoking and alcoholism are major risk factors (Chi et al., 2015; Li et al., 2016). Oral cancer caused by long-term use of tobacco is largely attributed to tobacco-specific nitrosamines. Alcoholic beverages may contain a variety of carcinogens and aldehydes, which can metabolize to acetaldehyde in the body (a proverbial carcinogen). Innutrition may also increase the risk of head and neck squamous cell carcinoma (HNSCC) in alcoholics. Notably, the combination of smoking and drinking shows a synergistic effect (Chi et al., 2015). Human papillomavirus is also a prime risk factor (Huang et al., 2008). The frequency of traditional risk factors for oral cancer, including tobacco and alcohol consumption, has recently declined (Warnakulasuriya, 2009). However, the incidence of head and neck tumors associated with human papillomavirus infection has increased and continues to rise globally (Chaturvedi et al., 2011; Jemal et al., 2013). Patients with immunosuppression are at the highest risk of developing oral cancer (Petersen, 2009). Immunosuppressive drugs appear to significantly contribute to the development of skin cancer/lip cancer and oral cancer after organ transplantation. This may be the result of immunosuppression or specific carcinogenic mechanisms. Chewing betel nut is also one of the dominant risk factors for oral cancer; of note, the betel nut itself is carcinogenic (Chi et al., 2015). In current clinical practice, the main treatment methods for oral cancer are surgery, chemotherapy, and radiotherapy. However, these methods are characterized by limitations. For example, surgery may cause damage to the shape and function of the patient’s head and neck, affecting their quality of life; mandibular resection damages the continuity of the mandible (Shah and Lydiatt, 1995). Radiotherapy may cause permanent xerostomia and radiation caries. Moreover, its therapeutic effect is limited by the development of radioresistance (Ishigami et al., 2007). The disadvantages of chemotherapy include non-specific biodistribution and multidrug resistance (Pérez-Herrero and Fernández-Medarde, 2015; Zhang et al., 2020a).

The current chemotherapeutic drugs used to treat oral cancer are cisplatin (DDP) (Pendleton and Grandis, 2013; Jiang et al., 2020), fluorouracil (5-FU) (Vodenkova et al., 2020), docetaxel (Cui et al., 2020), paclitaxel (Harada et al., 2014), and methotrexate (Zhu et al., 2017). The oral route is the best approach for the administration of drugs to the body. The advantage of oral administration is that it can improve the compliance of patients and prolong the exposure time of cytotoxic drugs; hence, it is suitable for outpatients (Terwogt et al., 1999). The limitations of oral administration are its poor water solubility, low bioavailability, and high toxicity (Devalapally et al., 2007; Agüeros et al., 2009).

Nanoplatform drug delivery systems were developed to overcome these problems. Nanoparticles (NPs) are solid colloidal particles composed of natural, synthetic, or semi-synthetic polymers with sizes ranging 1–1,000 nm (Kaur et al., 2004). Drugs can be dissolved, embedded, wrapped, or attached to the NP matrix, acting as a reservoir for the particle system and as a carrier for the drug delivery system, particularly in oncology (Cho et al., 2008; Couvreur, 2013; Feng et al., 2020). Currently, there are already nano-preparations for oral cancer treatment (Table 1).Compared with the traditional therapy of oral cancer, use of the nanoplatform may enhance the bioavailability and biodistribution of drugs in the original tumor site, shorten the treatment duration, and improve drug selectivity, thus reducing medical costs and improving patient compliance (Lu et al., 2018). On the one hand, NPs are passively targeted to the tumor site through their enhanced permeability and retention effect. On the other hand, the modification of active targeting molecules on NPs also helps in achieving effective drug delivery, leading to an improved therapeutic effect (Matsumura and Maeda, 1986; Chen et al., 2017; Zhang et al., 2018; Feng et al., 2019). For example, Endo et al. studied DDP-loaded polymer NPs NC-6004.Although the inhibitory effect of DDP on tumor cell growth *in vitro* is greater than that of NC-6004, they exert almost the same inhibitory effect on tumor growth *in vivo*. In addition, unlike mice injected with NC-6004, mice treated with DDP showed severe nephrotoxicity. This situation occurs because the polyethylene glycol (PEG)ylatedpolylactic-co-glycolic acid (PLGA) confers stealth properties to the formulation. This reduces the clearance of NC-6004 by the reticuloendothelial system, thereby prolonging the blood circulation time. The enhanced permeability and retention, as well as the prolonged blood circulation time lead to accumulation of DDP in tumor tissues. Wang et al. developed PEG-stabilized NR7 peptides and DDP-coupled PLGA NPs. NR7-PLGA NP-DDP has good characteristics, namely the targeting tumor cells, stability, high cell uptake rate, lower IC50 than free DDP and PLGA NP, and an excellent apoptotic effect. Compared with non-targeted PLGA NP, targeted PLGA NP can transport more DDP to cancer cells. In short, the NR7-PLGA NP-DDP system can be used as a cell-targeting nanoplatform for the treatment of oral cancer (Wang et al., 2015).Compared with traditional chemotherapeutic drugs, those based on nanoplatforms can achieve higher intra-tumor drug concentration and lower concentration in normal tissues. This has solved numerous problems (e.g., low oral bioavailability, non-specific biological distribution, and significant toxic reactions of traditional chemotherapy), and resulted in innovative changes to the drug treatment of oral cancer (Endo et al., 2013).

**TABLE 1:** The current research and development status of nano-preparations for commonly used oral cancer therapeutics.

Name of drug	Mechanism of action	Marketed preparation	System	Status	References
Cisplatin	Cisplatin can cross-link with DNA, destroy the function of DNA and prevent its repair, and inhibit cell mitosis	Lipoplatin	Liposome	Phase III clinical trial	Dilruba and Kalayda (2016)
Doxorubicin	Intercalation between the base pairs of the DNA strands, thus inhibiting the synthesis of DNA and RNA in tumor cells; production of iron-mediated free radicals, causing oxidative damage to DNA, proteins and cellular membranes	Anti-EGFR immunoliposomes	Liposome	Phase II clinical trial	Mamot et al. (2012), Lee et al. (2017)
Paclitaxel	Paclitaxel promotes mitotic stagnation and cell death by combining with microtubules, accelerating microtubule assembly, and maintaining tubulin polymers unchanged	Abraxane	Polymer NP	Approved internationally	Sofias et al. (2017)
Docetaxel (DTX)	DTX induces apoptosis by promoting the polymerization of microtubules, arresting the transition from metaphase to late stage, and initiating the spindle assembly checkpoint	DTX-SPL8783	Dendrimer	Phase I clinical trial	Kesharwani and Iyer (2015)
Vincristine	Vincristine is a microtubule-destabilizing agent, which affects microtubule dynamics, inducing abnormal mitotic spindle formation and causing cell arrest in the M phase and, subsequently, cell apoptosis	Vincristine sulfate liposome injection (VSLI)	Liposome	Phase II clinical trial	Schiller et al. (2018)

In this review, we introduce the nanoplatforms used for the treatment of oral cancer, and comprehensively compare the merits and demerits of these nanoplatforms (Table 2). These nanoplatforms are usually divided into the following categories: Polymer NPs, Lipid-based NPs, Metal NP, Hydrogels, Exosomes, and Dendrimers.

**TABLE 2:** The strengths and limitions, major characteristics, and composition of the nanoplatform in the treatment of oral cancer.

Nanoplatforms	Components	Strengths	Limitions	Characteristic	References
Polymer NPs	Indocyanine green, human serum albumin, cisplatin	Chemotherapy and PTT/PDT synergistic treatment	Low encapsulation rate	Actively target oral cancer with high expression of secreted protein acidic and rich in cysteine (SPARC)	Wang et al. (2019b)
Polymer NPs	PLGA, PEG, NR7 peptide	Core–shell morphology, excellent biodegradability	Early recognition by the immune system and clearance by the liver and kidneys limit it clinical application	Due to NR7 peptide receptor-mediated internalization, cancer cells uptake of nanoparticles increased	Pendleton and Grandis (2013)
Polymer NPs	Fucoidan, PI3Kα inhibitor BYL719	Combined with radiotherapy, nanoparticle administration can enhance anti-tumor activity without causing major side effects	Recognition and elimination by the immune system	The cell adhesion molecule P-selectin has nanomolar affinity for fucoidan, so nanoparticles can actively target cancer cells	Mizrachi et al. (2017)
Polymer NPs	HN-1 peptides, PEG, dox	The HNPD nanoplatform has strong tumor targeting performance and penetration efficiency	Low encapsulation rate	The PD nanoparticles synthesized by dox and PEG are a simple and effective nanocarrier	Wang et al. (2017)
NLC	Docetaxel (DTX),NLC	DTX can be well incorporated into NLC with high entrapment efficiency due to its lipophilicity	Lack of ability to actively target target cancer cells	Increase the drug loading efficiency and prolong the half-life of drug	Liu et al. (2011)
Liposome	Cationic liposome, adenoviral vector	Cationic liposomes combined with adenovirus vectors can improve gene transduction efficiency	Immune clearance and clinical application safety still need further research	Suicide gene therapy	Fukuhara et al. (2003)
Liposome	Anionic lipid, cationic lipid, cisplatin	The combined PDT + LPC prolonged the tumor growth inhibition, resulting in the minimal drug administrations	The ability of this nanoplatform to actively target tumor cells needs to be proven	Combined application of liposome-loaded chemotherapy and photodynamic therapy	Gusti-Ngurah-Putu et al. (2019)
Metal NP	Hollow gold nanospheres, aptamer targeted to EGFR	Gold nanospheres have excellent photodetection properties and can be used for imaging. Aptamers that target EGFR have high specificity and low immunogenicity	The phototherapy effect of hollow gold nanospheres is not fully utilized, and the photothermal therapy should be further explored; toxicity	Chemiluminescence optical imaging and RNA aptamer targeting EGFR	Melancon et al. (2014)
Metal NP	PEGylated AuNPs, PDPN Ab, dox	The tumor homing ligand on this nanoplatform can actively target cancer cells, deliver drugs to cancer cells, and cooperate with photothermal therapy to kill cancer cells	The early recognition of the immune system; short blood circulation and toxicity	Application of chemotherapy and photodynamic therapy	Liu et al. (2020)
Metal NP	Super paramagnetic iron oxide,PLGA, folic acid, chitosan	The nanoplatform can rapidly release docetaxel under acidic conditions and can avoid docetaxel leakage under physiological pH	The encapsulation rate of docetaxel in this nanoplatform is not clear	The magnetic iron oxide in the nanoplatform can be used in magnetic resonance imaging	Shanavas et al. (2017)
Metal NP	Polyacrylic acid, hollow mesoporous iron oxide, bleomycin	Magnetic nanoparticles are safe, non-toxic and can actively target cancer cells	The encapsulation efficiency of bleomycin in this nanoplatform is not clear; physiological pH releases more drugs than acidic pH	Surface-engineering polyacrylic acid (PAA) onto the mesoporous iron oxide makes the nanoplatform continuously release bleomycin under the magnetic field	Zhang et al. (2020b)
Hydrogels	Poly (ethylene glycol)-poly (ε-caprolactone)-poly (ethylene glycol) (PEG-PCL-PEG, PECE) hydrogel, cisplatin, suberoylanilide hydroxamic acid (SAHA)	The nanoplatform can be administered within a target organ at a predetermined rate and within a predetermined time, which reduces the drug poisonousness and improves the survival quality of patients	Elimination of nanoplatforms by immune cells	Temperature sensitive and injectable	Li et al. (2012)
Exosomes	Exosomes secreted by menstrual mesenchymal stem cells	Exosomes are nano-sized vesicles that produce therapeutic effects through paracrine action, and have long-term blood circulation and immune escape	The exosome extraction process is complicated and the number of exosomes obtained is limited-a fact that complicates translation of exosome treatments into the clinic	Exosomes are vesicles with a diameter of 40–100 nm, which have the inherent ability to cross biological barriers, even the blood brain barrier	Rosenberger et al. (2019)
Dendrimers	Polyamidoamine (PAMAM) dendrimer, folic acid	The well-defined and highly branched structure of dendrimers provides great flexibility for modification in terms of delivery of a large payload of drug and cell-specific targeting	Large-scale synthesis of functionalized dendrimers is technically challenging and potentially hinders their clinical applications	The surface-functionalized PAMAM dendrimer of folic acid reduces generation-dependent toxicity of PAMAM dendrimer, but it is still more efficient in gene delivery	Xu et al. (2016), Yuan et al. (2019)

## Research Progress in Nanoplatforms for the Treatment of Oral Cancer

### Polymer Nanoparticles

Polymer-based NPs are submicron-sized polymer colloidal particles in which the therapeutic agent of interest can be embedded or encapsulated in their polymer matrix, or adsorbed or bound to the surface. This type of NPs can improve the efficacy, solubility, toxicity, bioavailability, and pharmacokinetic properties of drug molecules, and deliver biomolecules, drugs, genes, and vaccines to specific targets (Mahapatro and Singh, 2011). Simultaneous application of photodynamic/photothermal therapy (PDT/PTT) can also improve the accuracy of the light position and extend the duration of drug action. Hu et al. synthesized an indocyanine green (ICG)-DDP coordination compound, and encapsulated ICG-DDP into human serum albumin (HSA) to form hybrid NPs (HSA-ICG-DDP NP). Using 808 nm laser irradiation, the coordination bond of ICG and DDP in HSA-ICG-DDP NPs is thermally cleaved, and DDP of HSA-ICG-DDP NPs is released from the cytoplasm (Figure 1). Hence, DDP accumulates in specific tumor sites, reducing the non-specific distribution of platinum. In addition, the coordination bond of ICG-DDP is broken due to the photothermal effect of ICG induced by near-infrared (NIR) radiation. DDP is accurately released at the specific tumor site under 808 nm NIR irradiation, thereby prolonging its action time. Therefore, HSA-ICG-DDP NPs are promising pre-clinical drugs for the PTT/PDT chemotherapy of OSCC (Wang et al., 2019b).

The polymer nanoplatform can reduce the non-specific distribution of chemotherapeutic drugs, and provide targeted drug delivery. The targeting specificity of polymer NPs with active targeting moieties has been previously reported. Wang et al. designed a polymer self-assembled NP. Based on the comparison of the tripeptide motif and the epidermal growth factor receptor (EGFR)-binding domain, the NP uses PLGA-PEG as a carrier and selects the NR7 peptide (NSVRGSR) to actively target specific tumor sites (Wang et al., 2015). DDP forms a cross-strand in DNA, which interferes with the ability of cancer cells to read or copy their genome, leading to programmed cell death (apoptosis) (Pendleton and Grandis, 2013). Although DDP has potential therapeutic effects, it is also linked to numerous serious side effects. The most common ones are gastrointestinal reactions, including nausea, vomiting, diarrhea. The administration of metoclopramide, dexamethasone, or ondansetron during the administration process can inhibit or reduce digestive tract reactions. Nephrotoxicity is the most serious toxic reaction. It is characterized by hematuria and renal damage, increased serum creatinine levels, and decreased clearance (Brillet et al., 1994; Arany and Safirstein, 2003; Wang et al., 2019a). PLGA-PEG nanoplatforms enable the more selective accumulation of DDP in tumors, while reducing its distribution in normal tissues. In addition, the NR7-targeting moiety exists on the surface of the PLGA carrier, which can achieve specific receptor-mediated internalization, while increasing cell uptake and lethality. It has been shown that the uptake rate of HN6 OSCC cancer cells was significantly increased, and a better anti-cancer effect was observed after optimized treatment with specific polymer NPs.

Mizrachi et al. added BYL719 (a PI3Kα inhibitor) to P-selectin-targeted NPs, which allowed it to accumulate specifically in cancer cells. The gene phosphatidylinositol-4,5-bisphosphate 3-kinase catalytic subunit alpha (PIK3CA), which encodes the phosphatidylinositol 3-kinase p110α subunit (PI3Kα), is frequently altered in HNSCC. PI3Kα inhibitors show good activity in a variety of cancers; however, their use is hindered by the side effects of dose limitation. P-selectin exists on the Weibel–Palade body membrane of vascular endothelial cells and platelet alpha-granule membrane. The cell adhesion molecule P-selectin has nanomolar affinity for fucoidan. Therefore, the embedded nano-PI3Kα inhibitor contains a polysaccharide polymer, while reducing the dosage and side effects of the drug. The drug targets specific cancer cells, thereby maintaining a good therapeutic effect (Mizrachi et al., 2017).

Wang et al. developed HN-1-modified PEGylated doxorubicin (HNPD) NPs, which are spherical, uniform in size, and have strong tumor-targeting properties and penetration efficiency. Owing to these characteristics, HNPD NPs specifically accumulate at the tumor site. This can enhance the therapeutic effect of DOX and reduce its toxicity. HNPD NPs can also slowly release DOX, extending its blood circulation time, and have good stability in the body. In addition, they release DOX *in vitro* with pH sensitivity. Compared with the control group, HNPD NPs have a higher cell uptake rate and cytotoxicity. Moreover, the tumor volume of tumor-bearing nude mice injected with HNPD NPs was smaller than that of control mice. Collectively, HNPD NPs can target tumor cells, exert good *in vivo* and *in vitro* therapeutic effects, and are simple to prepare. Hence, these novel nanoplatforms show potential for application in clinical practice (Wang et al., 2017).

### Lipid-Based Nanoparticles

**FIGURE 2:**
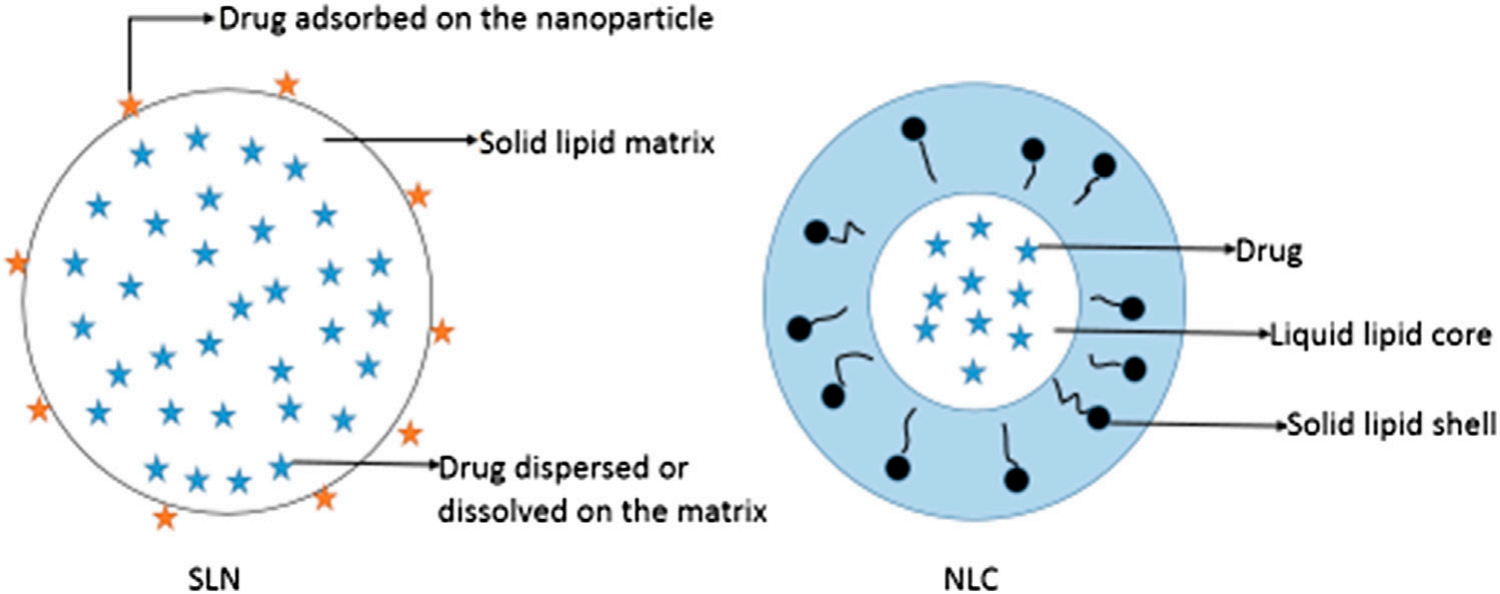
Schematic diagrams of solid lipid nanoparticles (SLNs) and nanostructured lipid carriers (NLCs).

Lipid-based NPs include SLNs, NLCs and liposomes. SLNs are a relatively new class of drug carriers. They are particles of submicron size (50–1,000 nm) and composed of lipids that remain in a solid state at room temperature and body temperature; of note, drugs can be dissolved or dispersed in solid lipids (Wong et al., 2007) (Figure 2). SLNs exhibit physical stability, protect unstable drugs from degradation, control drug release, and are associated with good tolerance (Wissing et al., 2004; Souto and Doktorovová, 2009; Mu and Holm, 2018). Pindprolu et al. prepared STAT3 inhibitor niclosamide (Niclo) SLNs (CD133-Niclo-SLNS) modified with CD133 aptamers. Niclo exhibits poor water solubility, it is easily removed, and its low bioavailability limits clinical application. SLNs are suitable for the packaging of poorly soluble drugs and can be used as a carrier for intravenous injection or local administration to achieve targeted positioning and controlled release. Moreover, they can be used for the packaging of Niclo to improve the stability and performance of the drug. In addition, CD133 aptamers can be used as effective targeting ligands to deliver drugs to CD133 cancer stem cells. The prepared SLNs (CD133-Niclo-SLNS) are stable, and can actively target tumor cells to prevent stem cells and epithelial cell-mesenchymal transition-mediated recurrence (Pindiprolu and Pindiprolu, 2019).

SLNs also have inevitable limitations. High-pressure homogenization is commonly used in the preparation of SLNs. However, the high temperature reached during this process accelerates the degradation rate of drugs and lipids. The coexistence of gelation and other colloidal structures, drug precipitation, particle size growth, and kinetic phenomena are disadvantages of SLNs. Solid lipids are mixed with liquid lipids of different shapes to prepare a new generation of lipid NPs-NLC (Fang et al., 2008; Battaglia and Gallarate, 2012). NLCs are composed of solid lipids enclosing variable liquid lipid nanocompartments (Figure 2). The addition of liquid lipids disrupts the regular lattice structure of solid lipids, increases the proportion of irregular crystal forms in the NP structure, increases the space capacity of the NPs, and improves the drug-carrying capacity. Liquid lipids are controlled by the surrounding solid lipid barrier. Therefore, NLCs can maintain a solid skeleton structure at body temperature to achieve controlled release of NLC drugs (Fang et al., 2008; Lin et al., 2010; Kovacevic et al., 2011). Liu et al. designed DTX-NLC with stearic acid, monoglyceride, soybean lecithin, and oleic acid as the main raw materials, and prepared DTX-NLC using an improved thin-film ultrasonic dispersion method. DTX is fixed in the lipid core of NLCs and can be released for a long time, reducing the number of administrations. In addition, DTX-NLC exhibits a stronger cytotoxic effect than free DTX. This may be because DTX-NLC NPs enter cancer cells through endocytosis, which enhances the accumulation of drugs in cells (Liu et al., 2011). NLCs provide targeted delivery, which improves the treatment efficacy of anti-cancer drugs and reduces their side effects. Therefore, as a carrier, NLCs can provide anti-tumor drug targeting and intracellular administration (Fang et al., 2013).

Liposomes are formed by lipid bilayers and a water core layered by cholesterol. They can encapsulate water-soluble and non-water-soluble drugs in lipid bilayers to form microcapsules with variable sizes (Hu and Zhang, 2012).Liposomes have attracted considerable attention as a valuable carrier and drug delivery system owing to their high drug loading capacity and the flexibility of photosensitizers adapted to different physical and chemical properties. Compared with free drugs, liposome preparations can be released slowly, reduce the drug’s poisonousness on cells, andlengthen the action time of the drug, thereby showing better anti-tumor activity.Liposomes can also encapsulate water-soluble and hydrophobic drugs into selected tissues in a rate-controlled release manner (Derycke and de Witte, 2004; Krajewska et al., 2019). It has been reported that liposomes can mediate gene transduction to treat oral cancer. Konopka et al. used polycationic liposomes as the carrier of DNA for gene therapy of HNSCC. Studies have found that polycationic liposomes can mediate gene transduction under high fetal calf serum conditions, and have a lower carrier immune response. Therefore, in a specific biological environment, polycationic liposomes can be used for gene delivery (Konopka et al., 2005). Fukuhara et al. investigated the treatment of oral cancer by liposomes, and evaluated the effects of a new cationic liposome-coupled adenovirus vector (Ad/SUV) on the gene transduction efficiency of four human oral cancer cell lines and one mouse squamous cell carcinoma cell line. Synthetic Ad/SUV can enhance gene transduction to human oral cancer cells and kill tumor cells. The reason for this phenomenon is that liposomes reduce the neutralization of adenovirus vectors by antibodies. The gene transduction efficiency of Ad/SUV and the killing effect on tumor cells are obviously stronger than that of Ad vector alone. In short, the novel cationic liposome-coupled Ad carrier has strong anti-tumor activity on human OSCC (Fukuhara et al., 2003).

PEGylatedadriamycin liposome (Doxil) is a particular dosage form of adriamycin composed of monolayer liposomes. Methoxy PEG is encapsulated on 1,2-distearoylglycerol-3-phosphoethanolamine and exists on the inner and outer surfaces of the lipid bilayer. Owing to its water solubility, doxorubicin (DOX) is stably encapsulated in the water core of liposomes (Mamidi et al., 2010). Doxil is approximately 100 nm in size and can be selectively delivered to tumor sites, permitting it to infiltrate deficient blood vessels in tumor sites. El-Hamid et al. studied the effectiveness of adriamycin and its nanoform (Doxil) to induce apoptosis in oral cancer CAL-27 cells. Compared with the necrosis of cancer cells caused by adriamycin, Doxil mainly exerts its therapeutic action by inducing apoptosis in cancer cells. Doxil-treated cells showed 3.38-fold higher caspase-3 levels than control cells, while free DOX-treated cells showed 2.72-fold higher caspase-3 levels than control cells. The percentage of C-Myc mRNA inhibition in Doxil-treated was higher than that observed in DOX-treated cells. In summary, Doxil induced apoptosis in CAL-27 cells to a greater degree than DOX (El-Hamid et al., 2019).

**FIGURE 3:**
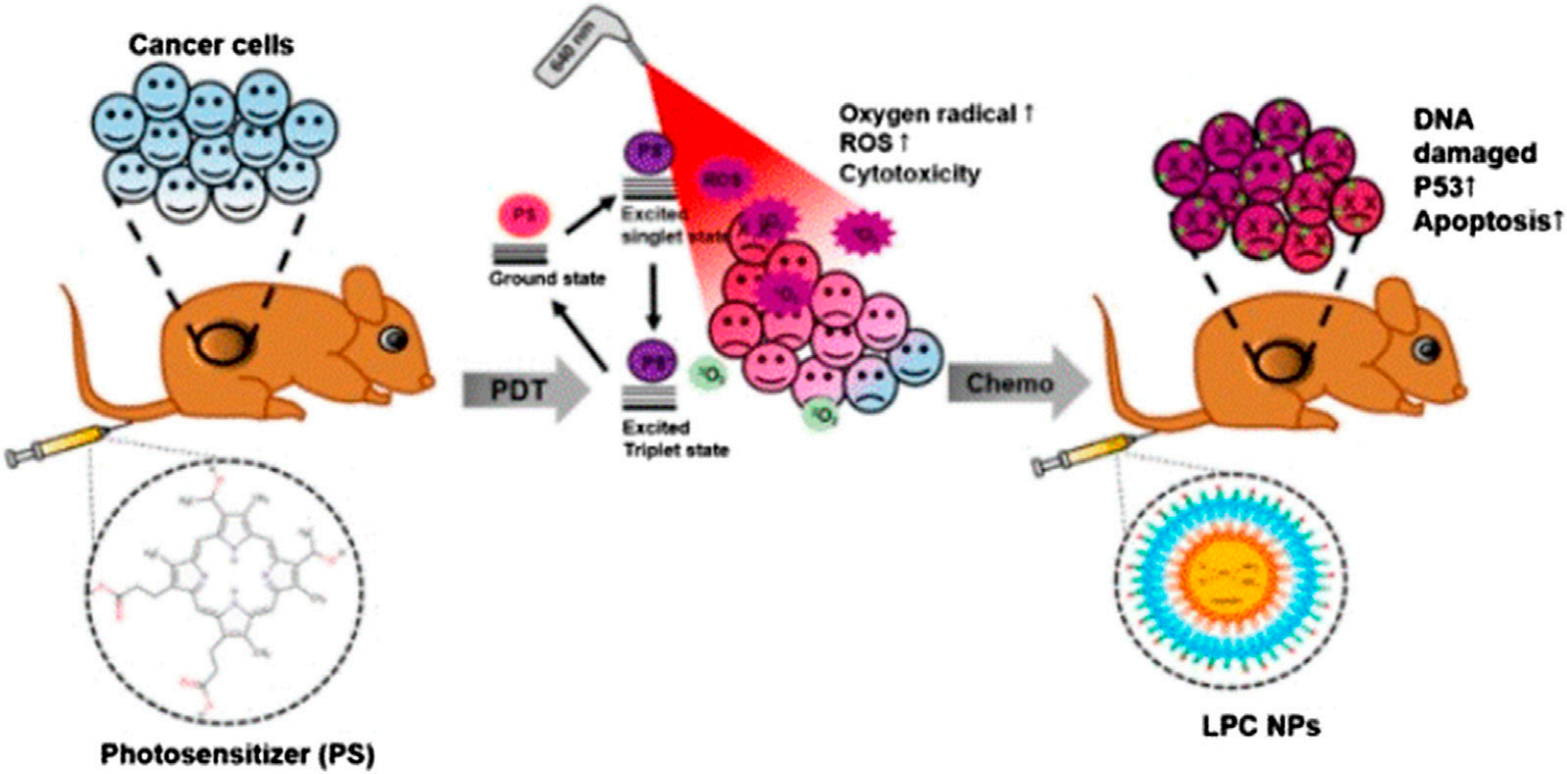
Schematic diagram of *in vivo* study using LPC NP and PDT combination therapy. Reproduced from (Gusti-Ngurah-Putu et al., 2019)with permission from Journal of Clinical Medicine.

Lipid-platinum-chloride (LPC) NP is a nanoplatform formed by liposomes loaded with DDP, and exerts a significant tumor suppressor effect in many types of cancer (Guo et al., 2014a; Guo et al., 2014b; Putra et al., 2016). LPC has unique characteristics, including instantaneous release of platinum for 3-4 h and adjacent effect characteristics (Guo et al., 2013). Eka-Putra et al. reported the therapeutic effect of PDT + LPC on an OSCC xenograft model (Figure 3). The results showed that PDT + LPC can fully reduce the tumor volume by 112%. Tumor volume in the LPC, PDT + DDP, and DDP groups was reduced by 98.8%, 73.1%, and 39.5%, respectively. Histological examination showed that, compared with the DDP or PDT + DDP group, treatment with PDT + LPC or LPC had the least toxic effects on kidneys. Immunohistochemical staining, TUNEL detection, and immunoblotting of tumor suppressor gene p53 verified these findings. Above all, LPC + PDT extended the inhibition of tumor growth, reducing the requirement of chemotherapy. Therefore, treatment with PDT and LPC NPs has a positive therapeutic effect on human oral cancer (Gusti-Ngurah-Putu et al., 2019).

Mohan et al. developed and characterized PEGylated liposome nanocarriers wrapped with *trans*-resveratrol and adriamycin. Both drugs are contained in liposomes, and the supreme encapsulation efficiency of each drug is approximately 80%, when the ratio of resveratrol to DOX is 2:1. The liposome nanoplatform offers slow drug release, decreases the toxicity of the drug to normal tissue, and augments drug concentration at the tumor site, thus showing higher anti-tumor activity vs. free drugs. In addition, the liposome nanoplatform also regulates the cell cycle and downstream proteins, leading to apoptosis of cancer cells. This study revealed the application prospect of liposomes as nanoplatform carriers in the treatment of oral cancer (Mohan et al., 2016).

Longo et al. found that a liposomal aluminum-chloro-phthalocyanine (AlClPc) preparation combined with PDT caused necrosis in Ehrlich tumor cells in the tongue of Swiss mice with strong immunity. The average diameter of liposomal AlClPc is between 120 and 200 nm, easily penetrating into tumor blood vessels. This results in a higher number of passive accumulations of nanoplatforms in tumor tissues vs. normal tissues. The combination of hydrophobic photosensitizers and liposomes preferentially induces cell death through necrosis. The preferred target of this nanoplatform is the phospholipid cell membrane. Photoactivation of the photosensitizer located at this site causes rupture of the cell membrane and destruction of other organelles, leading to cell death. Approximately, 90% of tumor necrosis is attributed to the synergistic effect of liposomal AlClPc-mediated direct toxicity after PDT and tumor vessel closure. Therefore, liposomal AlClPc-mediated PDT is effective in treating oral cancer (Longo et al., 2009).

### Metal Nanoparticles

Metal NP includes Au NP and MNP. AuNPs exhibit plasmon resonance and have a highly specific surface area, which enables the modified AuNPs to load drug, thereby improving the solubility, stability, and pharmacokinetic parameters of the drug. Owing to the characteristic photonic properties of AuNPs (surface plasmon resonance absorption and resonance light scattering), their applications in the biological and medical fields are particularly attractive. Preliminary research has investigated the application of nano-gold as a biomedical contrast agent in confocal scanning optical microscopy (Sokolov et al., 2003), multiphotonplasmon resonance microscopy (Yelin et al., 2003), and optical coherence microscopy (Raub et al., 2004). In addition, AuNPs also have many advantages: ease of detection; inert; lack of toxicity; high scattering intensity; and higher brightness than chemical fluorophores. Due to their supramolecular structure, AuNPs are useful for detecting, diagnosing, and treating tumors. It is an effective reagent used for the determination of heavy metal ions, as well as DNA and protein analysis; it is a chemotherapy carrier for the transport of biomolecules and drug molecules (Kumar et al., 2013).

Coupling antibodies to AuNPs can make NPs actively target cancer cells, which is useful in revealing the internal function of cancer cells and producing better therapies. In addition, by using intelligent bio-coupling technology (Jiao et al., 2011), AuNPs can be functionalized with different molecules. Thus, they are capable of executing targeted, diagnostic, and treatment functions in a single treatment process. This type of multifunctional NP has been used in exalting applications to *in vivo* and *invitro* therapy experiments (Liang et al., 2014). Biscaglia et al. prepared PEG-bare AuNPs modified with lysine and Ge11. They found that these NPs possess better targeting properties compared with AuNPs modified with cetuximab (C225) (Biscaglia et al., 2017). Melancon et al. prepared hollow gold nanospheres that encapsulate an aptamer targeting EGFR. The hollow gold nanospheres are connected with single-stranded DNA. Subsequently, EGFR-targeting RNA complementary to the single-stranded DNA is added, so that the hollow gold nanospheres have the ability to target EGFR-positive cancer cells. It has been shown that hollow gold nanospheres modified with ^111^In have a more obvious selective killing effect on EGFR-positive cancer cells than those labeled with an anti-EGFR antibody (C225). Moreover, the physical and chemical properties of the hollow gold nanospheres did not change after modification. This shows that hollow gold nanospheres, as a carrier for a new nanoplatform, can stably and accurately transport the aptamer to EGFR-positive oral cancer cells, providing a promising new direction for the treatment of oral cancer (Melancon et al., 2014).

**FIGURE 4:**
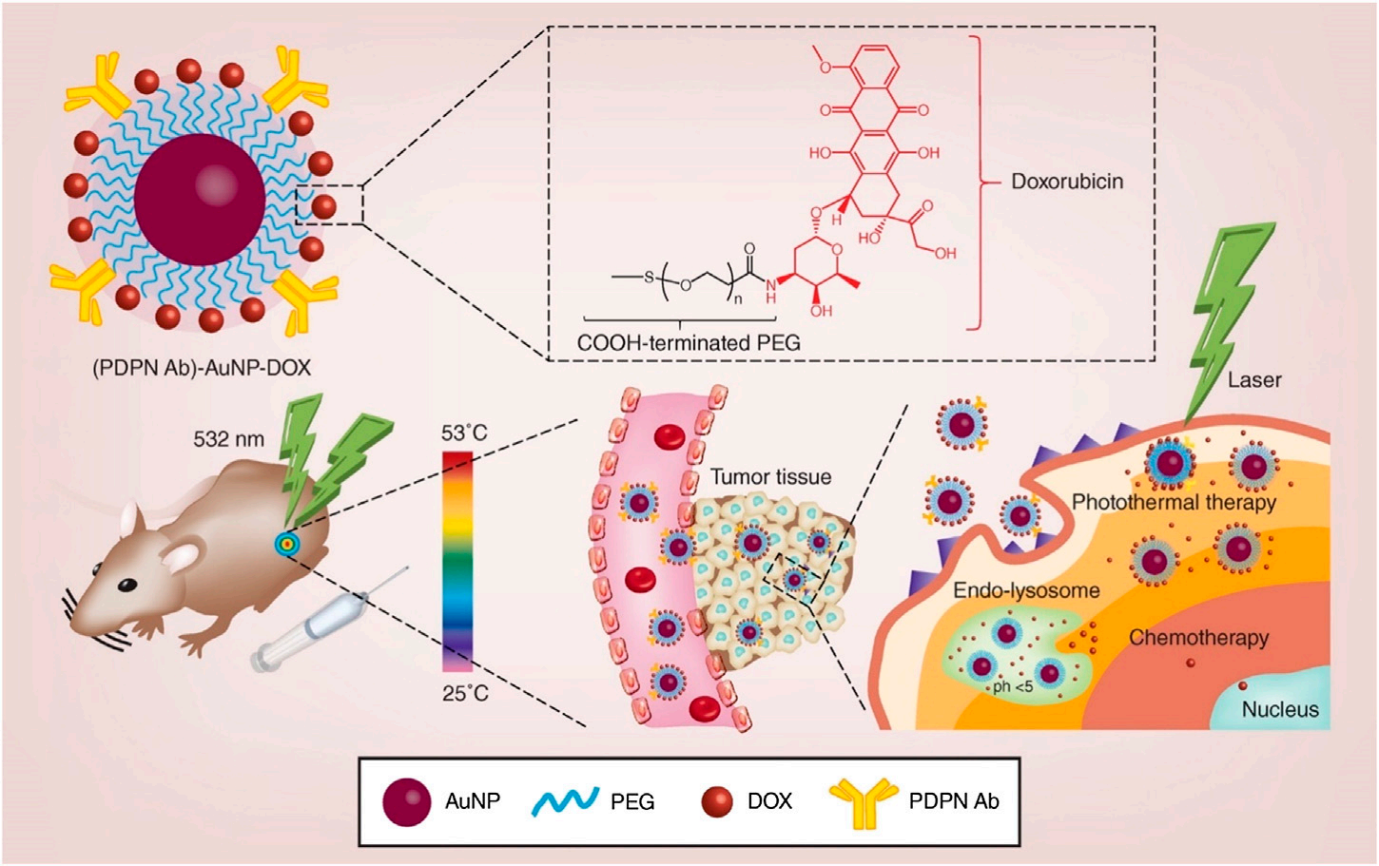
Schematic illustration of the synthesis and application of PDPN antibody-gold nanoparticle-doxorubicin for chemo-photothermal cancer therapy. AuNP: gold nanoparticle; DOX: doxorubicin; PDPN Ab: podoplanin antibody; PEG: polyethylene glycol. Reproduced from (Liu et al., 2020) with permission from *Nanomedicine (Lond)*.

In another study, Liu et al. prepared PEG-stabilized podoplanin antibody (PDPN Ab) and DOX-coupling AuNPs (Figure 4). (PDPN Ab)-AuNP-DOX has good biocompatibility, drug loading capacity, cell uptake efficiency, pH-dependent drug release characteristics, far lower half maximal inhibitory concentration (IC50) than free DOX, and higher photothermal conversion efficiency. Following laser irradiation, (PDPN Ab)-AuNP DOX exerts enhanced anti-tumor effects *in vivo* and *in vitro*. The (PDPN Ab)-AuNP-DOX system serves as a multifunctional combined chemotherapy/PTT nanoplatform for the treatment of oral cancer (Liu et al., 2020).

Reza et al. grafted anti-human epidermal growth factor receptor 2 (anti-HER2) nanoantibodies to gold-silica nanoshells, and used the optical properties of gold to trigger photothermal treatment (PTT) with NIR light for the killing of oral cancer cells. The prepared nanoplatform was co-cultured with KB epithelial cells and HeLa cells (control group) excited with NIR light; this was followed by detection of photothermal toxicity. It was found that the number of KB tumor cells that died was relatively large, whereas there was almost no cell damage or death noted in the HeLa cells. The higher number of KB tumor cell deaths is mainly related to the positive HER2 on the surface, which makes the nanoplatform selectively accumulate in tumor cells. Thus, labeling NPs with antibodies can improve their targeting properties; this approach has become a new method for the treatment of oral cancer (Fekrazad et al., 2011).

AuNPs can be used as carriers for the delivery of drugs to tumor cells, and have therapeutic effects. Essawy et al. compared two nanostructures coupled with DOX by means of a pH-sensitive and pH-resistant linker. The results of *in vitro* experiments showed that the pH-resistant DOX nanostructure exerted a greater cytotoxic effect in HSC-3 cells compared with pH-sensitive DOX AuNPs. The former has a long-term cytotoxic effect, whereas the latter shows a short-term effect. In addition, the stably connected DOX nanostructure were found to induce cancer cell death through apoptosis, while the DOX AuNPs trigger a necrotic reaction. These results indicate that the stable DOX nanostructure can induce powerful cell death. The results of *in vivo* experiments showed that tumor shrinkage and the survival rate of animals treated with DOX pH-resistant AuNPs were significantly improved compared with those recorded in animals treated with the pH-sensitive type. These *in vitro* and *in vivo* data strongly indicate that AuNPs have greater potency as drug transporters (Essawy et al., 2020).

For superficial tumors (e.g., HNSCC), magnetic drug targeting has achieved a good therapeutic effect. As one of the most promising materials, MNPs are non-toxic to humans, and have been used as a basic platform for imaging, targeted drug delivery, and monitoring efficacy. As one of the most prospective nanomedicine carriers, superparamagnetic NPs (under the control of an external magnetic field) can specifically concentrate the drugs on the lesions, thereby minimizing treatment-related side effects (Laurent et al., 2014; Singh and Sahoo, 2014; Wang and Gu, 2015; Siafaka et al., 2016).

In view of the large number of reports on ferric oxide NPs, folic acid, chitosan, and PLGA in the biomedical field, use of these materials is important for the careful design of NPs to utilize their great advantages and obtain nanomaterials with excellent properties. Using magnetic PLGA nanoparticle as “core” and folic acid-chitosan conjugated as “shell” to modify the surface, Shanavas et al. prepared magnetic core-shell hybrid NPs through the nanoprecipitation method. Using the best molar ratio of folic acid to amine (chitosan), the folic acid-chitosan conjugate was prepared by the carbodiimide cross-linking chemical method, and further coated on the magnetic PLGA NPs encapsulated with docetaxel. The magnetic PLGA nanoplatform modified by folic acid-chitosan is a kind of hybrid NP with a core-shell structure. Folate-positive KB cells can bind to folic acid on their surface and selectively uptake the nanoplatform. The nanoplatform targets cancer cells through folate receptors and plays an anticancer role. In addition, because the protonation of chitosan on nanoparticle surface reduces the resistance of drug release, the nanoplatform can rapidly release docetaxel under acidic conditions. Under physiological pH, folic acid-chitosan can control docetaxel release and avoid drug leakage. The magnetic iron oxide in the nanoplatform can be used in magnetic resonance imaging. It can be observed that the magnetic PLGA hybrid NPs modified by folic acid-chitosan are promising nanoplatforms with good biocompatibility, and can be used in the magnetic resonance imaging and treatment of cancer (Shanavas et al., 2017).

**FIGURE 5:**
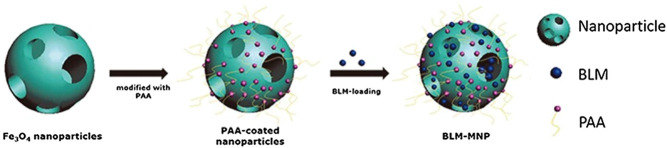
Schematic showing the synthesis of the magnetic nanoparticle with surface-engineering PAA in the outer layer and BLM molecules bonded with PAA. BLM: Bleomycin PAA: Polyacrylic acid Reproduced from (Zhang et al., 2020b) with permission from American journal of cnacer research.

Zhang et al. announced the preparation and functionalization of biocompatible superparamagnetic hollow mesoporous NPs. The surface engineering of polyacrylic acid is processed on the superparamagnetic NPs that can support bleomycin in the mesoporous structure, and bonded with polyacrylic acid to construct a nanoscale drug delivery system (Figure 5). The drug is targeted through the nanoplatform, stays in the focal area under the magnetic field, and is continuously released. Detailed studies have shown that polyacrylic acid functionalized MNPs loaded with bleomycin can stimulate local tumor cell apoptosis. This nanoplatform endows anti-cancer drugs with targeting ability *in vitro* and inhibits tumor development *in vivo* (Zhang et al., 2020b). Lu et al. developed a nanoplatform composed of pH-dependent β-cyclodextrin and magnetic colloidal NPs. Compared with individual magnetic nanocrystals, β-cyclodextrin and magnetic nanocrystal composites have a higher loading rate of 5-FU (Lv et al., 2014). Anirudhan et al. used chemical precipitation to prepare a maleic anhydride-grafted magnetic cyclodextrin derivative to control the release of 5-FU. The synthetic compound has a good safety profile, high water solubility, and high pH sensitivity. Experimental results regarding the function of this nanoplatform in breast cancer therapy showed that the cytotoxicity of cyclodextrin-MNP was markedly enhanced compared with that of the 5-FU control group. The MNP-CD nanoplatform has low toxicity and side effects on normal cells (Anirudhan et al., 2015).

Miao et al. combined poly (ethylene imine) (PEI)-modified iron oxide NPs with the human telomerase reverse transcriptase (hTERT) promoter to form a new nanoplatform. This nanoplatform can deliver the hTERT promoter to tumors, and activate the human tumor necrosis factor-related apoptosis-inducing ligand (TRAIL) gene to cause apoptosis in oral cancer cells. Under the action of a magnetic field, the PEI-modified Fe_3_O_4_ MNPs are positively charged, whereas the hTERT promoter is negatively charged. Through the combination of the two under the action of a magnetic field, PEI modification can protect DNA from digestion by endosomes and improve the transfection efficiency. In the experimental group, 73% of the tumor cells were positive for TRAIL staining, whereas the tumor cells in the control group were negative. This study showed that PEI-modified iron oxide NPs are promising nanoplatforms that can be used to deliver gene therapy for oral cancer (Miao et al., 2014).

### Hydrogels

Hydrogels have adjustable physical and mechanical properties, and can be widely used in the medical field. In this regard, they have been used as drug delivery systems for several years because they provide a convenient support matrix for the active ingredients (Drury and Mooney, 2003). For example, Li et al. successfully developed a biodegradable thermosensitive hydrogel that can be loaded with succinimidylhydroxamic acid (SAHA) and DDP. The nanoplatform can be administered within a target organ at a predetermined rate and within a predetermined time, which overcomes the shortcomings of traditional pharmaceutical preparations. This reduces the drug poisonousness and improves the survival quality of patients. In this study, mice xenotransplanted with HSC-3 cells were classified into six groups, and were infected with saline, SAHA, poly (ethylene glycol)-poly (ε-caprolactone)-poly (ethylene glycol) (PEG-PCL-PEG, PECE) hydrogel, DDP, SAHA-DDP, or SAHA-DDP/PECE. Compared with other control groups, the two combined treatment groups (particularly the SAHA-DDP/PECE treatment group) had significantly reduced tumor growth. The cell apoptosis rate of the combined treatment group was significantly higher than that of the control group, and the tumor volume was the smallest. These results showed that the SAHA-DDP/PECE nanoplatform can effectively inhibit the development of oral tumor cells. Therefore, PECE hydrogel-mediated DDP and SAHA may become a novel and promising chemotherapy for oral cancer (Li et al., 2012).

### Exosomes

Exosomes are vesicles with a diameter of 40–100 nm, which can be separated from cell culture supernatants and different biological fluids. They can be captured by neighboring recipient cells through the interaction of vesicle surface ligands and cell receptors, and subsequently fuse with recipient cells through internalization (Théry et al., 2006; Théry et al., 2009). Exosomes include many types of biomolecules; thus, they play a significant role in intercellular communication (Bunggulawa et al., 2018). Studies have shown that exosomes belonging to extracellular vesicles can target diseased tissues or organs (Wiklander et al., 2015). Most cells can secrete exosomes, but some cells can actively secrete them, such as macrophages (Bhatnagar et al., 2007), B cells (Clayton et al., 2005), T cells (Nolte-'t Hoen et al., 2009), mesenchymal stem (Lai et al., 2015), endothelial (Song et al., 2014), and epithelial cells (Skogberg et al., 2015).

They are highly effective drug carriers that can provide cell-based drug delivery. Exosomes or exosome-like vesicles can passively load small lipophilic molecules and large molecules (e.g., DNA, RNA, and proteins) into exosomes. The surface proteins of exosomes allow their load to easily pass through the cell membrane and deliver their contents in a biologically active form. More importantly, exosomes have the inherent ability to cross biological barriers, even the blood brain barrier (Batrakova and Kim, 2015).

**FIGURE 6:**
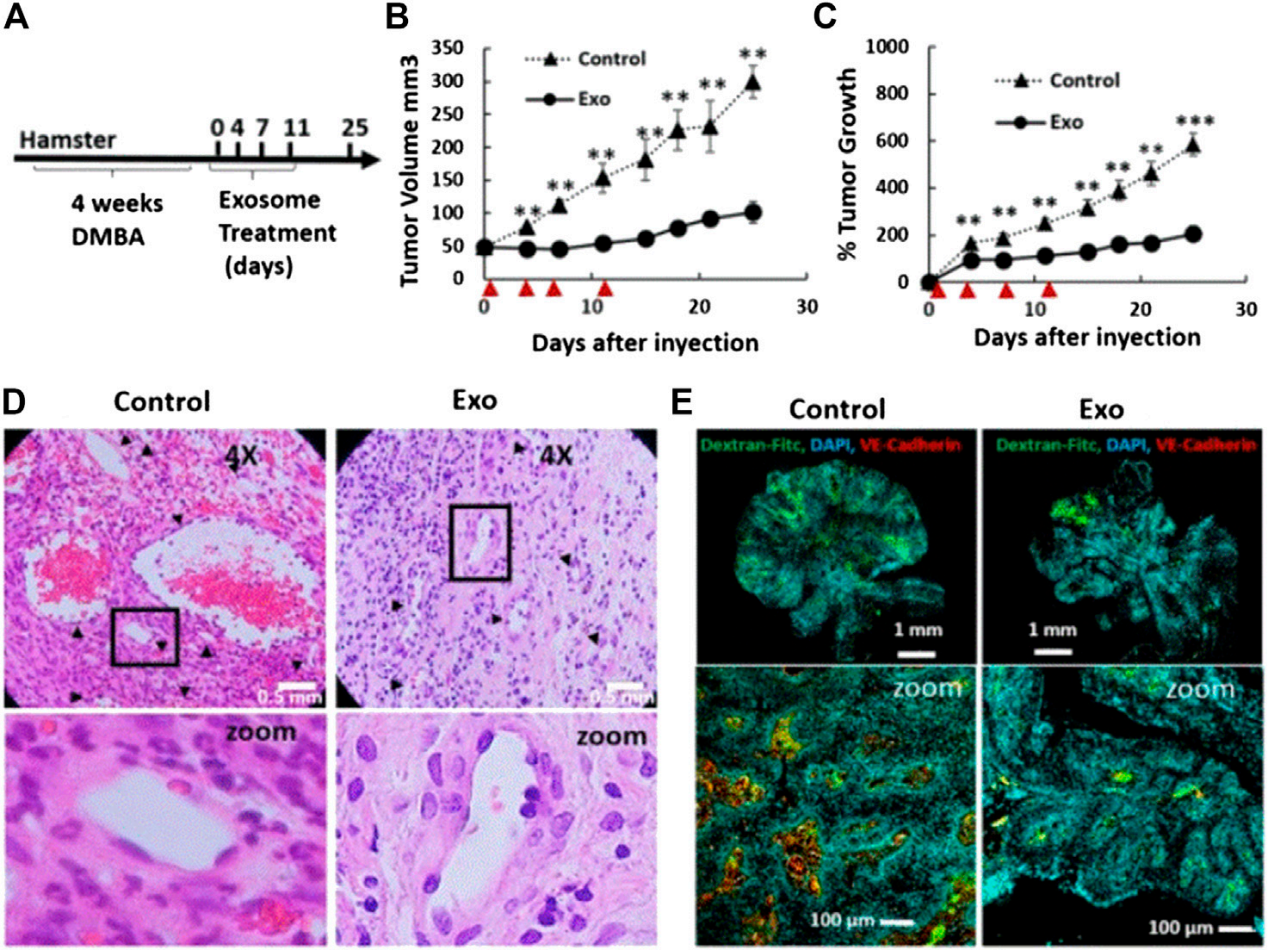
Tumor growth and angiogenesis is conspicuously restrained by exosome therapy. **(A)** Scheme of experimental design. DMBA injection induced tumor production within 4 weeks, and exosomes were injected every 3–4 days for a total of four injections. **(B and C)** Tumor growth and volume of the control and exosomes groups after four injections of exosomes. ▲ indicates days of exosome treatment. **(D)** H&E sections of tumor tissues in the control and exosome treatment group on day 25. **(E)** Histological sections of tumors stained with Hoechst (blue), Dextran-FITC (green), and VE Cadherin (red) at day 25. Reproduced from (Rosenberger et al., 2019) with permission from *Scientific Reports*. DAPI, 4′,6-diamidino-2-phenylindole; DMBA, 7,12-dimethylbenzanthracene; FITC, fluorescein; isothiocyanate; H&E, hematoxylin-eosin; VE, vascular endothelial.

Research studies found that exosomes can be loaded with chemotherapeutic drugs to treat OSCC. Rosenberger et al. investigated the therapeutic effect of menstrual mesenchymal stem cell (MenSC)-derived exosomes on hamster buccal pouch carcinoma, and confirmed that intratumoral injection of MenSC-exosomes leads to significant anti-tumor effects and tumor blood vessel loss. They found that the biological effects of MenSC-exosomes on endothelial cells and their anti-angiogenic effects may have advantages in the treatment of OSCC. Moreover, they also proposed a method to expand the production of exosomes using the fiber-based microcarrierBioNOC II, which can reduce the production cost. It is established that endothelial cells are responsible for angiogenesis. In this study, following the evaluation of cytotoxicity, they found that exosomes induce endothelial cell death, which may be one of the reasons for tumor blood vessel loss. In addition, they also assessed whether MenSC-exosomes can directly regulate the angiogenic potential of endothelial cells. The results showed that the anti-angiogenic effect of MenSC-exosomes is a unique feature of these exosomes, and is not necessarily attributed to other cell types. More importantly, the anti-angiogenic property of MenSC-exosomes exerts a significant effect on hamster cheek pouch carcinoma. As shown in Figures 6B,C, after four injections of exosomes in hamsters with cheek pouch cancer, it was found that the tumor volume and growth were reduced compared with those recorded in the control group. As illustrated in Figures 6D,E, compared with the control group, the tumor blood vessel density and blood vessel area of the exosome treatment group were significantly reduced. This evidence shows that exosomes can also inhibit OSCC *in vivo*. In summary, owing to their biological effects on endothelial cells and anti-angiogenesis, exosomes may become a promising nanoplatform for the treatment of OSCC (Rosenberger et al., 2019).

### Dendrimers

The use of nano-level therapy has many advantages compared with the existing methods for the treatment of human diseases. In these nanoplatforms, the well-defined and highly branched structure of dendrimers provides great flexibility for modification, especially for cell targeting, high-dose drug loading, gene therapy payloads, or their combinations (Yuan et al., 2019). Dendrimers can couple alternative targeted drugs and therapeutic drugs to a single carrier device. Dendrimer-based methods are exploring many alternative targets, which may allow us to modify therapies (receptor targeting and therapeutic utilization) based on the genetic makeup of tumor characteristics in the future (Ward et al., 2011).

Xu et al. developed a folic acid-modified polyamidoaminedendrimer G4 (G4-FA) nanoplatform for the targeted delivery of DNA plasmids to head and neck cancer cells that highly express folate receptors. G4-FA has good cell compatibility and can contend with free folic acid for identical binding sites on cancer cells. G4-FA can specifically bind to folate receptors to accelerate the uptake of DNA plasmids by cancer cells, and selectively deliver the plasmids to cancer cells with high expression of the folate receptor to increase gene expression (Xu et al., 2016).

Particularly, Xu et al. designed a folic acid-modified polyamidoaminedendrimer as a carrier to deliver siRNA. Following modification of the nanoplatform with folic acid, it can be absorbed by tumor cells that highly express folic acid receptors through endocytosis mediated by these receptors. SiRNA targeting vascular endothelial growth factor A enters tumor cells through endocytosis, reducing the molecular targets responsible for tumor cell proliferation and survival, and exerting anti-cancer effects. The G4-FA nanoplatform mainly increases its concentration in tumors through endocytosis, thereby maintaining a high siRNA concentration in the tumor to inhibit growth. According to the evaluation of NIR imaging, G4-FA injected into the tumor showed a high tumor absorption rate and sustained high local retention. Both single-dose and double-dose G4-FA/vascular endothelial growth factor A (siVEGFA) can inhibit tumors. However, the tumor volume increased on day 8 in the single-dose group. This phenomenon disappeared after the application of the double dose, indicating that the double dose may have a sustained anti-tumor effect. In short, G4-FA is a safe nanoplatform that can specifically deliver siRNA to locally target the treatment of head and neck cancer (Xu et al., 2017).

## Conclusion

Chemotherapy remains the main treatment strategy for patients with oral cancer. Considering the various shortcomings of this therapeutic approach, nanoplatforms are being developed. In this review, we have summarized a variety of nanoplatforms for the treatment of oral cancer. These nanoplatforms can overcome many shortcomings of chemotherapy, enable drugs to accurately reach tumor cells, reduce side effects on surrounding normal tissues, and bring hope for the discovery of new oral cancer therapies.

However, In order to apply the nanoplatforms for the clinical treatment of oral cancer, several barriers, such as the controversial EPR effect, toxicity and instability, insufficient blood circulation time, need to be overcome (Blanco et al., 2015). Many nanoplatforms target specific tumor cells through the EPR effect. However, studies have shown that the variability of the EPR effect in large animals or humans has rarely been considered (Hansen et al., 2015). The short blood circulation of some nanoplatforms makes them cleared by the mononuclear phagocyte system (MPS) and reticulo-endothelial system (RES) (Pérez-Herrero and Fernández-Medarde, 2015). In addition, toxicity and instability also limit the clinical application of nanoplatforms. AuNPs provoke an imbalance in the oxidative status of the cells, which is accompanied by damage in the genetic, lipid and protein structures. Therefore, it is strongly recommended to conduct a deeper study about the use of AuNPs as drug delivery vehicle in the chronic treatment of diseases as cancer (Lopez-Chaves et al., 2018). Due to the premature release of drugs and serious accumulation or misplaced aggregation, the poor colloidal stability of nanoplatforms always leads to low drug delivery efficiency (Sang et al., 2019).

In order to improve the performance of nanoplatforms for oral cancer therapy, some factors should be considered. To start with, the change in the nanoplatforms’ size may lead to a different nanomedicine physiological stability. A suitable size of nanoplatforms is absolutely necessary to enhance their therapeutic effect (He et al., 2019). Secondary, modifying the surface properties of nanoparticles with cell membranes to prolong blood circulation time and immune escape (Chen et al., 2016). Meanwhile, the design of appropriate clinical trials is crucial. Furthermore, investigators should also design some nanoplatforms that are easy to use in the clinic, and design animal models for *in vivo* experiments.

## Author Contributions

All authors have made a substantial, direct and intellectual contribution to the work, and approved it for publication.

## Conflict of Interest

The authors declare that the research was conducted in the absence of any commercial or financial relationships that could be construed as a potential conflict of interest.
